# Antibody-Dependent Cellular Phagocytosis in Antiviral Immune Responses

**DOI:** 10.3389/fimmu.2019.00332

**Published:** 2019-02-28

**Authors:** Matthew Zirui Tay, Kevin Wiehe, Justin Pollara

**Affiliations:** ^1^Department of Molecular Genetics and Microbiology, Duke University School of Medicine, Durham, NC, United States; ^2^Human Vaccine Institute, Duke University School of Medicine, Durham, NC, United States; ^3^Department of Surgery, Duke University School of Medicine, Durham, NC, United States

**Keywords:** antibody effector functions, antibody-dependent cellular phagocytosis (ADCP), Fc receptors, phagocytes, antiviral antibodies

## Abstract

Antiviral activities of antibodies may either be dependent only on interactions between the antibody and cognate antigen, as in binding and neutralization of an infectious virion, or instead may require interactions between antibody–antigen immune complexes and immunoproteins or Fc receptor expressing immune effector cells. These Fc receptor-dependent antibody functions provide a direct link between the innate and adaptive immune systems by combining the potent antiviral activity of innate effector cells with the diversity and specificity of the adaptive humoral response. The Fc receptor-dependent function of antibody-dependent cellular phagocytosis (ADCP) provides mechanisms for clearance of virus and virus-infected cells, as well as for stimulation of downstream adaptive immune responses by facilitating antigen presentation, or by stimulating the secretion of inflammatory mediators. In this review, we discuss the properties of Fc receptors, antibodies, and effector cells that influence ADCP. We also provide and interpret evidence from studies that support a potential role for ADCP in either inhibiting or enhancing viral infection. Finally, we describe current approaches used to measure antiviral ADCP and discuss considerations for the translation of studies performed in animal models. We propose that additional investigation into the role of ADCP in protective viral responses, the specific virus epitopes targeted by ADCP antibodies, and the types of phagocytes and Fc receptors involved in ADCP at sites of virus infection will provide insight into strategies to successfully leverage this important immune response for improved antiviral immunity through rational vaccine design.

## Introduction

**Figure 1:**
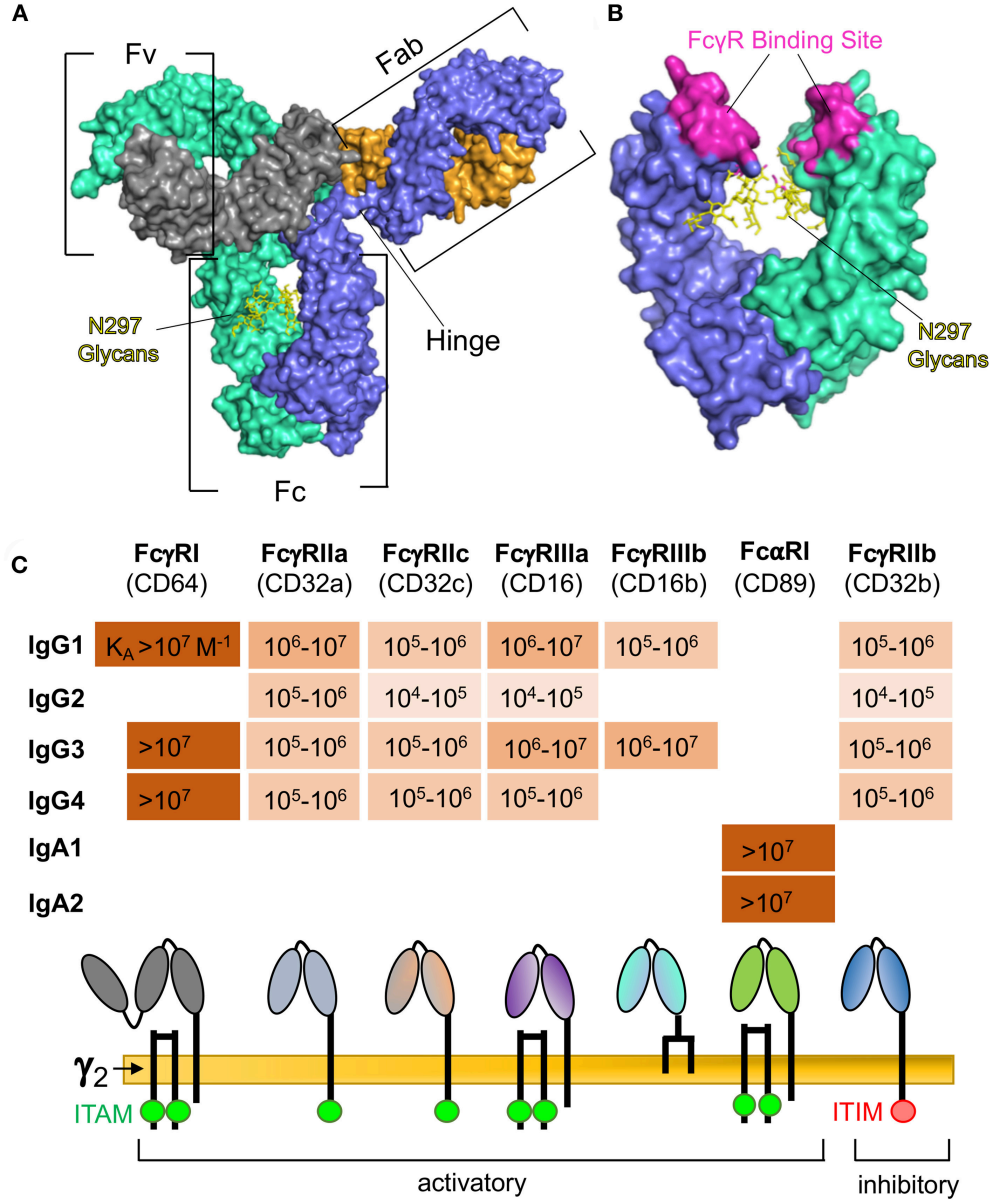
**(A)** Surface representation of human IgG1 indicating: the Fv regions, which are the portions of the Fab arms required for antigen binding; the Fc region, which interacts with immunoproteins and specific receptors for effector functions; and the hinge region, which provides flexibility for the Fab and Fc regions. The IgG heavy chain 1 is depicted in blue, heavy chain 2 in teal, light chain 1 in orange, and light chain 2 in gray. Glycans are represented by yellow sticks. Rendering was made in PyMOL software using protein data bank ID 1HZH. **(B)** Expanded view of human IgG1 Fc region after 180° rotation, indicating residues involved in binding to Fcγ receptors (2–5), and the glycosylation site at asparagine 297 (N297). **(C)** Binding affinities (6–8) of Fc receptors commonly implicated in ADCP and comparison of receptor composition and signaling domains. Absence of values indicates no detectable binding.

Antibodies are a key component of the human adaptive immune system, and the elicitation of antibodies has been correlated with vaccine efficacy in many diseases (1). Passively infused antibodies have been used in anti-toxin, anti-viral, and anti-inflammatory treatments; and monoclonal recombinant antibodies are also currently being pursued for prevention of HIV-1 infection in large Phase IIb clinical trials (NTC02716675 and NCT02568215). Antibodies can exert their protective functions via a multitude of mechanisms. Some functions, such as neutralization, mainly depend on interaction of the Fv domain (Figure 1A) with antigen and are therefore predominantly Fc domain independent. Other functions, including antibody-dependent cell-mediated cytotoxicity (ADCC) and antibody-dependent cellular phagocytosis (ADCP), require interactions between the antibody Fc domain with other proteins or immune effector cells via recognition by Fc receptors (9–11) (Figures 1A–C). These Fc receptor-dependent antibody functions provide a direct link between the innate and adaptive immune systems, harnessing the potent anti-pathogen functions of the innate immune system, and overcoming its inherent limited pattern recognition capacity by utilizing the diversity and specificity of the adaptive immune response. Fc receptor-dependent antibody functions are important components of the immune response that provide mechanisms for clearance of infected host cells, immune complexes, or opsonized pathogens. Fc receptor-dependent antibody functions are also involved in activation of downstream adaptive immune responses by facilitating antigen presentation or by stimulating the secretion of inflammatory mediators (12, 13). This review is focused on the antibody Fc receptor-dependent effector function ADCP in immune responses against viruses and targets three areas of interest: (1) discussion of the biophysical factors that influence ADCP including the properties of the receptors, antibodies, and effector cells; (2) survey and interpretation of evidence supporting a potential role for ADCP in either inhibiting or enhancing viral infection; and (3) description of current approaches used to measure ADCP with consideration for the translation of studies performed in animal models.

## Antibody and Fc Receptor Interactions Involved in ADCP

Immune complexes formed between antigen and antibody are capable of engaging a diversity of Fc receptors on innate immune cells. The type I IgG Fc receptors are activatory FcγRI, FcγRIIa, FcγRIIc, FcγRIIIa, FcγRIIIb, and inhibitory FcγRIIb (Figure 1C). Other IgG Fc receptors include the non-classical (type II) IgG Fc receptors CD209 and CD23, neonatal FcR (FcRn) which is involved in IgG transport and recycling, and the cytosolic Fc receptor TRIM21. IgA antibodies are specifically engaged by FcαRI (Figure 1C), and the specific receptor for IgE is FcεRI—which is involved in rapid allergic responses. Immune complexes may also interact with other receptors that have been described to bind to immunoglobulins but have been relatively uncharacterized, including FCA/MR (14), FCMR (15), IgD-R (16), CD71 (17), secretory component receptors (18), asialoglycoprotein receptors (19), and M cell receptors (20).

When engaged, most Fc receptors are capable of cytoplasmic signaling. For instance, as shown in Figure 1C, FcγRIIa and FcγRIIc signal via their immunoreceptor tyrosine-based activation motif (ITAM) domains, whereas FcγRI and FcγRIIIa lack ITAM domains but associate with an FcRγ signaling chain (γ_2_) and signal via its ITAM domain. The inhibitory FcγRIIb signals via an immunoreceptor tyrosine-based inhibition motif (ITIM) domain. The IgA receptor, FcαRI, also associates with the FcRγ signaling chain (Figure 1C), but this seems to be dispensable for signaling, and signaling is dependent on dephosphorylation of the intracellular domain of FcαRI (21). Downstream signaling pathways are complex and dependent on the Fc receptor, cell type, and stimulation mechanism, but generally act via increasing intracellular calcium cation concentration, activation of PKC, or activation of ras (22).

**Figure 2:**
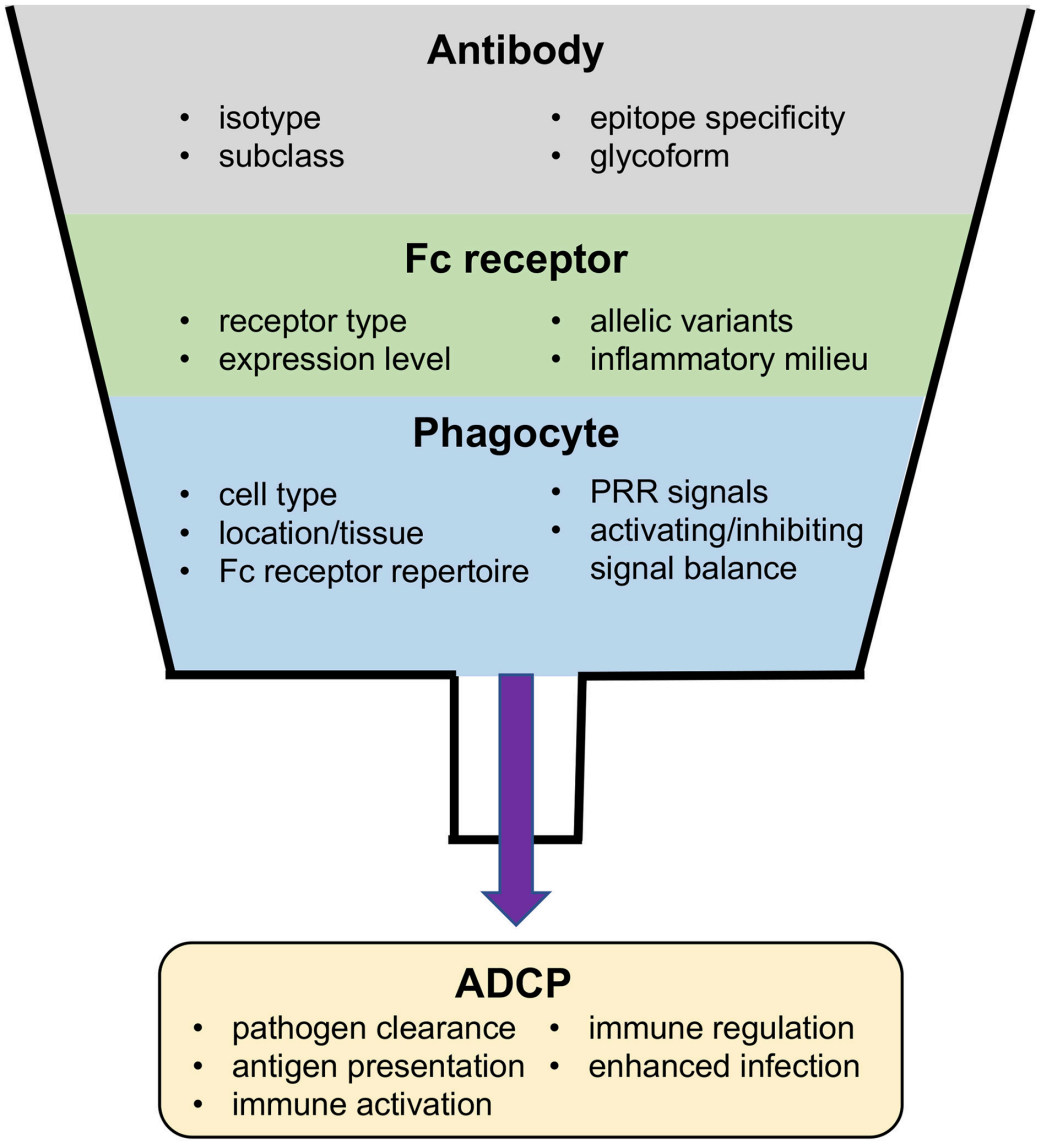
Factors impacting ADCP and potential outcomes of the ADCP response to virus infection.

As a result of the ubiquitous presence of antibody in both the systemic and mucosal microenvironments, regulatory systems are required to prevent constitutive Fc receptor signaling. This is achieved via several mechanisms that are either intrinsic to Fc receptor signal pathways, impacted by external soluble signals detected by the phagocyte, or established at the genetic level (Figure 2). A key intrinsic regulator is the inability of free antibody to activate Fc receptor signaling. The low-affinity Fc receptors, including FcγRIIa, FcγRIIb, FcγRIIc, and FcγRIIIa require multiple coordinated interactions for sufficient binding avidity, and thus can only be triggered by multivalent antibody-antigen immune complexes (22). Even for the high-affinity FcγRI, which is able to bind monomeric IgG, binding does not trigger signaling through its associated γ-chain; instead, signaling requires receptor clustering and cross-linking (23). Moreover, activatory Fc receptors can also produce inhibitory signals when engaged at a low level–the mechanisms for this phenomenon are not well-understood but may involve ITAM monophosphorylation which activates the inhibitory SHIP-1 (24) rather than the diphosphorylation required for activatory Syk engagement (25). Similarly, immunostimulatory and immunoinhibitory Fc receptors are often co-expressed on the same cell, thus the outcome of antibody-mediated signaling is often dependent on the balance of activating or inhibiting signals. The abundance of these receptors on the cell-surface and their ability to interact with immune complexes is influenced by soluble signaling molecules, which allows the local inflammatory milieu to also contribute to the regulation of Fc receptor-dependent responses of phagocytes (26–28). Finally, Fc receptor signaling is regulated at the genetic level. Single nucleotide polymorphisms in human Fc receptors affect interactions with antibody Fc, resulting in Fc receptor variants with lower or higher relative affinities for immune complexes (2, 6, 11, 12, 29). Several Fc receptor polymorphisms have been associated with the occurrence or progress of disease resulting from infection with viruses including dengue virus (30, 31), influenza virus (32), human coronavirus (33), Epstein-Barr virus (EBV) (34), Kaposi's Sarcoma virus (KSV) (35), and HIV-1 (36–38).

The specific Fc receptor engagements involved in ADCP of different viruses or virus-infected cells at sites of infection and throughout antiviral immune responses have not been completely defined, and are not expected to follow a generalizable rule. As will be described in the following sections, the receptors involved will differ depending on the characteristics of the antibody forming the complex —such as isotype, subclass, and glycosylation— as well as on the particular type of phagocyte encountering the immune complex.

## Antibody Characteristics That Impact ADCP

In addition to the Fc receptor regulatory mechanisms described above, the ability of interactions between immune complexes and Fc receptors to result in Fc receptor signal transduction is further regulated by specific characteristics of the antibody Fc region. Antibody isotype serves as the principal level of regulation, as most Fc receptors are specific for only one isotype of antibody. Within isotype, there is additional regulation at the level of antibody subclass (Figure 1C). For the human IgG isotype, IgG3 has the highest affinity for most of the type I FcγRs, followed by IgG1, then IgG4, then IgG2 (6). In contrast, subclass is not a predominant source of regulation for ADCP by IgA, as human FcαR has been demonstrated to have similar affinity for IgA1, and IgA2 (7).

Additional regulation of Fc–Fc receptor interactions required for ADCP occurs via diversity in glycosylation of the antibody Fc (39). The IgG Fc region contains an N-linked glycan at asparagine 297 (Figure 1B) that impacts the conformation of the antibody and affinity for Fc receptors (39). As described in a comprehensive recent review by Jennewein and Alter, for IgG antibodies there are 36 possible glycoforms and 4 different subclasses, yielding a total of 144 possible unique Fc regions (40). Importantly, antibody glycovariation can be modulated by inflammatory responses, allowing the immune system to adapt and adjust antibody Fc glycoforms to modulate biologic activities in response to infection (40). Changes in antibody glycosylation have been described during the course of HIV-1 infection (41, 42), and in response to influenza vaccination (43, 44), but for many viral diseases the role of glycovariation in antiviral antibody responses has yet to be defined.

## Fc Receptor Signaling for ADCP

Leukocytes involved in ADCP must express at least one type of Fc receptor. This includes monocytes, macrophages, neutrophils, and eosinophils; canonically referred to as professional phagocytes. In ADCP the phagocyte is engaged by antibody, either directly via Fc receptor or indirectly via antibody-fixed complement, to engulf one or more opsonized particles or molecules, which typically including pathogens, infected cells, and their derivatives (45, 46). The internalization in most cases leads to the destruction of the internalized target by phagolysosomal degradation, though it is important to note that several human pathogens have evolved to co-opt this process and survive within phagocytes (47–50). Phagocytosis leads to different immune outcomes depending on the cell type—for instance, antibody-mediated phagocytosis by macrophages leads to enhanced pathogen destruction and antigen presentation, whereas antibody-mediated phagocytosis by plasmacytoid dendritic cells leads to enhanced secretion of interferon alpha (51–53).

In Fc receptor-mediated phagocytosis, ligated and aggregated Fc receptor become phosphorylated via Src family tyrosine kinases (54) on their ITAM domains (either their own or from an associated γ-subunit), forming a docking site for Syk (55) and triggering a signaling cascade involving PKC (56, 57), PI3K (58–60), and synthesis of PI(4,5)P2, (3,4,5)-PIP3, and DAG (61). These lead to actin cytoskeleton remodeling (62), allowing the advance of the phagocytic cell over the target. The strength of early signaling events is proportional to the number of engaged Fc receptors, whereas late signaling events required to complete phagocytosis require a concentration threshold of 3′PI to be satisfied (63).

## Phagocytes Involved in Virus ADCP

The type of phagocyte involved in an ADCP responses to virus infection depends not only on the profile of Fc receptors expressed by the cell and characteristics of the antiviral antibodies as described above, but also on the phagocyte being present at, or recruited to, sites of infection. Professional phagocytes are differentially distributed in the circulation and tissues (64–67), and inflammatory signaling can promote both ingress and egress of immune effector cells (68). Therefore, cells present at the site of infection and involved in the antiviral response likely change over time. Transgenic mouse models and cell-type specific depletions can help to identify essential cell populations, but a clear view of the specific interactions involved in the tissue at the site of virus infection is often limited, especially in humans. Despite the difficulties inherent to accessing and studying immune cells *in situ* within human tissues, a remarkable study by Sips et al. defined the distribution and frequency of Fc receptor expressing immune cells in mucosal and lymphoid tissues (69). They identified differential distribution of professional phagocytes —with macrophages being the dominant phagocyte population in lymph nodes, and intestinal tissues and neutrophils representing the dominant phagocyte population in tissues from the lower female reproductive tract. Using a novel tissue phagocytosis assay, they compared the HIV-1-specific ADCP activity of neutrophils and macrophages from the colon and cervix. They found that although abundant in the colon, colon-resident macrophages were deficient in ADCP compared to colon- and cervix-resident neutrophils as well as cervix-resident macrophages. This seminal study likely only partially defines the diversity of professional phagocytes, both for phenotype and functionality, within tissues that can be encountered by antibody-virus immune complexes during virus infection, and that inevitably impacts outcome of these encounters.

## Balance of Activatory and Inhibitory Signals Determine How Phagocytes Respond to Immune Complexes

Importantly, most phagocytes are capable of other Fc-dependent effector functions in addition to ADCP. Thus, the outcome of each interaction between phagocytes and immune complexes is determined by a combination of signals (Figure 2). Many phagocytes express more than one type of Fc receptor, often expressing both activatory and inhibitory Fc receptors. The balance of these divergent signal pathways is critical to simulating and regulating each potential effector response. Signals mediated by other types of receptors also contribute to determining the type of effector response a phagocyte will mount. Among them, information from pattern recognition receptors (PRRs) that can detect molecular patters associated with different types of pathogens are integrated in the response to Fc receptor stimuli. For example, Toll-like receptor 3, 7, 8, and 9 have the ability to detect viral nucleic acids and activate immune cells (70), and are therefore able to potentiate effective antiviral responses including ADCP. In contrast, alternative inhibitory signals, such as CD47 SIPα can negatively regulate phagocytosis. Intriguingly, some viruses express homologues of CD47, which may act to prevent activation of professional phagocytes as a strategy for immune evasion (71, 72).

Apart from direct antiviral activity through uptake and elimination of virus or infected cells, antibody-dependent phagocytosis is also important in the development and regulation of immune responses themselves (Figure 2) (12). Pathogen-associated molecular patterns (PAMPs) derived from virus antigens can be released upon phagocytosis and digestion of antibody–virus immune complexes. The released virus PAMPs can prime an inflammatory response upon sensing by PRRs, which may then stimulate additional immune cells and activate subsequent immune responses (12, 13). For instance, in adenovirus infection of the respiratory tract, alveolar macrophages are responsible for internalizing adenovirus and initiating early pro-inflammatory signaling (73). A similar response, termed antibody-induced inflammation, has been demonstrated to have an important role in protection against influenza infection (74). There are also direct roles for ADCP in modulation of adaptive immunity. ADCP of immune complexes by dendritic cells via FcγRIIa promotes MHC class I and II antigen presentation and induces cellular and humoral immune responses, while uptake through FcγRIIb prevents dendritic cell maturation and does not promote immune activation (75). Antibody production and affinity is also regulated by FcγRIIb-mediated induction of B-cell apoptosis, which helps to eliminate B cells with low affinity B cell receptors (75). Based on the diverse roles of ADCP in immune responses it is unsurprising that ADCP has been shown to be an important component of immune responses to infection by many different viruses.

## Evidence For the Importance of ADCP in Immune Responses Against Viruses

Phagocytosis has traditionally been known for its role in clearance of bacteria and fungi, as evidenced by the fact that persons with defects in phagocytosis are susceptible to common bacterial and fungal infections (76). It is important to note that these observations relate to total phagocytosis (antibody dependent and antibody independent) and thus do not allow for dissection of the specific contribution of ADCP to protection from bacterial and fungal infection. The role of ADCP in immune responses against viruses is similarly complex and difficult to dissect given its association with other antibody functions in the settings of infection, vaccination, and passive immunization.

### HIV-1

In the context of natural infection with HIV-1, the first line of evidence for a role of ADCP in the antiviral immune response is associations between Fc receptor genetics and disease progression or risk of infection. Forthal and collaborators (37) performed FcγRIIa genotyping of a large cohort HIV-1 infected men (*n* = 559), over 90% of which were entered into the study prior to the availability of antiretroviral therapy, and all of whom were enrolled into the cohort with CD4^+^ T cell counts above 500/mm^3^. They found that homozygosity for the low affinity allele of FcγRIIa (R/R131), the Fc receptor implicated in IgG-mediated ADCP activity of antibody responses against HIV-1 (77, 78), significantly predicted an accelerated rate of disease progression —defined as CD4^+^ T cell counts under 200/mm^3^–when compared to subjects that were heterozygous for this allele (H/R131), or homozygous for the high affinity allele (H/H131) of FcγRIIa (37). No such correlation was observed for FcγRIIIa allelic variants, which is conventionally regarded as the primary Fc receptor involved in natural killer (NK) cell ADCC. Associations between Fc receptor genetics and risk of HIV-1 infection have also been studied in the setting of vertical transmission. Using samples collected from antiretroviral-naïve HIV-seropositive mothers and paired infants in western Kenya, Brouwer et al. (36) identified infant homozygosity for the high affinity allele of FcγRIIa as a risk factor for perinatal HIV-1 infection. They observed no impact of maternal FcγRIIa alleles on transmission. Recently, a similar study was conducted using samples collected from 79 HIV-1 transmitting mothers, 234 non-transmitting mothers, and their offspring, in a South African cohort with contrasting results (79). In this latter study, the infant FcγRIIa acquisition risk factor identified by Brouwer et al. was not recapitulated, but instead the authors reported that mothers with the high affinity allele for FcγRIIIa (homozygous or heterozygous) were associated with a significantly lower risk of HIV-1 vertical transmission (79). Taken collectively, these three observations highlight the complexity in interpreting correlative studies. How can the seemingly contradictory findings be explained? Many factors are likely contributing to the divergent outcomes including differential requirements for immune control of disease progression vs. infection when comparing the Forthal study to the two studies investigating vertical transmission. Within the transmission studies, the authors of the South African study put forth the hypothesis that differences in timepoints used for determination of HIV infection may have resulted in inclusion of more postnatal breastfeeding transmission events in the Brouwer study, which likely have different requirements for protection compared to infections occurring in utero or perinatally. Although this may be the case, it is also important to reiterate that Fc receptor genetics is only one level of regulation for ADCP and other Fc receptor-dependent immune responses. As previously described, further regulation occurs at the level of the cell (type of phagocyte and combination of receptors expressed), within tissue immune environment (presence or absence of inflammatory signals), and via the antibody comprising the immune complex (specificity, isotype, subclass, and glycoforms). Thus, although studies based on genetic factors alone may provide insight into the potential for ADCP and other Fc receptor-dependent antibody functions to contribute to immune responses to HIV-1 they are limited by an inability to account for the myriad of factors that impact these antibody effector functions *in vivo*. Other studies have helped to address some of these limitations and provide additional evidence that ADCP plays an important antiviral role against HIV-1. By comparing ADCP activity of polyclonal IgG collected from HIV-1 infected individuals and healthy controls, Ackerman and collaborators determined that phagocytosis activity was higher for IgG from viremic patients and HIV-1 controllers compared to IgG collected from patients on highly active antiretroviral therapy. Importantly, they found that the antibodies from controllers were able to outcompete phagocytic activity of antibodies from viremic individuals and were biased toward interactions with activatory FcγRIIa over interactions with inhibitory FcγRIIb (78). In follow-up studies, they demonstrated that ADCP was a component of a polyfunctional response in HIV-1 controllers that included NK cell activation, ADCC, and complement deposition (80), and that was likely impacted by skewing of antibody glycoforms (42). Unlike that observed for HIV controllers, impaired phagocytosis is one of the hallmarks of chronic viremic HIV-1 infection (81–83), and may be related to a loss of FcγRII expression on monocytes and dendritic cells (77).

Preclinical and clinical trials of candidate HIV-1 vaccines have provided more opportunities to evaluate the importance of ADCP in protection against SIV/SHIV/HIV-1 infection. Immune correlates analyses suggest that protection against infection as well as inhibition of virus replication after establishment of infection is mediated not only by direct neutralization, but also by Fc-mediated antibody effector functions (80, 84–92).

In rhesus macaque SIV and SHIV preclinical animal models, non-broadly neutralizing antibody functions, including phagocytosis, correlated with reduced risk of infection as measured by increased number of low-dose challenges to infection (86, 90, 93). Of particular interest is the elegant study by Ackerman and colleagues, which identified distinct immune signatures of vaccine-mediated protection dependent on the route of immunization (94). In this study, rhesus macaques were immunized with a DNA prime-Ad5 SIVmac239 Env-based vaccine regime, either via the intramuscular (IM) route, or intranasally in an aerosol (AE) formulation. Equivalent levels of vaccine efficacy (~70%) against repeated low dose smE660 intra-rectal challenge were observed for both the IM and AE immunization groups, although unique humoral immune profiles and correlates of risk were identified. ADCP however, was identified as a correlate of reduced infection risk in both the IM and AE vaccine groups. Remarkably, although ADCP was a common immune function linked to protection independently of the route of immunization, the phagocytes and antibody isotypes associated with ADCP differed. For animals vaccinated by the IM route, monocyte ADCP and IgG were associated with reduced risk of infection, while ADCP by neutrophils (termed antibody-dependent neutrophil phagocytosis, ADNP) and IgA were associated with reduced risk of infection in animals vaccinated via the AE route. Importantly, a cross study validation of the ADCP correlate was preformed, and ADCP was also identified as associated with reduced risk of infection by low dose SHIV challenge in rhesus macaques vaccinated with an ALVAC prime gp120-boost vaccine regimen (93, 94), providing evidence for ADCP in vaccine-elicited protection afforded by different vaccine regimens and routes of inoculation, and against different challenge viruses.

Consistent with the observations from efficacious preclinical studies performed in rhesus macaque models, an immune correlates analysis of the partially efficacious RV144 vaccine human clinical trial provided evidence that non-neutralizing antibodies contributed to reduced risk of infection. Vaccine-elicited variable region 1 and 2 (V1/V2) IgG antibodies correlated with decreased risk of HIV-1 infection (85, 89, 91, 95) and these V1/V2 antibodies were not broadly neutralizing but were capable of multiple antiviral functions, such as ADCC, virion capture, ADCP, and tier-1 neutralization (91, 96–98). Notably, the RV144 vaccine regimen elicited antibodies that exhibited coordinated Fc-mediated effector responses (87, 91). Fc receptor polymorphisms also influenced RV144 vaccine efficacy (99), although these polymorphisms were for FcγRIIc and have not been associated with ADCP. Other HIV-1 vaccine efficacy trials that showed no efficacy either lacked a coordinated Fc receptor-dependent effector response (87) or lacked evidence of strong Fc-mediated antibody functions (100, 101).

When considered collectively, the results from non-human primate and HIV-1 candidate vaccine clinical trials provide strong evidence that ADCP is an achievable and potentially protective antiviral immune response to induce by preventative HIV-1 vaccines. As efforts continue toward development of vaccines that can induce broad-neutralizing antibodies it will be important to ensure that ADCP and other Fc receptor-dependent antibody responses are elicited. Fortunately, although HIV-1 broadly neutralizing antibodies (bnAbs) are defined based on their ability to neutralize a broad range of viruses, many bnAbs are also capable of mediating Fc receptor-dependent antiviral functions including ADCC and ADCP (69, 102–104).

Passive immunization trials have also show that antibody-mediated protective activity is not solely due to neutralization, but also in part due to Fc receptor-dependent functions. In non-human primate (NHP) passive immunization studies, with both high and low dose vaginal challenge of rhesus macaques with SHIV162p3, protection decreased by about 50% when the administered passive antibody was incapable of binding Fc receptors (105, 106). Similarly, in passive immunization studies performed with humanized mice, antibodies with enhanced ability to bind activating Fc receptors gave greater protection than their epitope-matched counterparts (107, 108). Although the specific role of ADCP in these observations has not been determined, when combined with the evidence provided from studies of HIV-1 virus control and disease progression, and from candidate vaccine trials, there is strong evidence to support ADCP as contributing to antibody-mediated protection from HIV-1. However, a recent study by Parsons and collaborators demonstrated that for the highly potent neutralizing antibody PGT121, Fc-receptor dependent functions were dispensable for maximal protection (109). These results suggest that the extent to which Fc receptor-dependent antibody functions contribute to protection is variable, and likely dependent on multiple factors including neutralization potency and/or characteristics of the virus challenge (110).

### Influenza Virus

Huber and colleagues used a murine vaccination and challenge model to provide evidence supporting a role of ADCP in protective immune responses to influenza virus infection (111). Normal BALB/c mice and BALB/c mice engineered to lack expression of the Fc receptor γ-chain—and thus deficient in Fc receptor signaling—were given identical influenza immunizations and challenges. Mice lacking the Fc receptor γ-chain were incapable of antibody-mediated phagocytosis, and were highly susceptible to influenza infection, despite the presence of normal levels of cytokines (IFN-γ and IL-10) and antibodies. The condition was not reversed even in the presence of passively-infused anti-influenza antibodies from immune wild type mice, indicating that a defect in interactions of antibody with immune effector cells caused the susceptibility. The effector mechanism was narrowed down by demonstrating protection after passive transfer of immune sera into CD3ε-transgenic mice that lack T cells and NK cells, thereby showing lack of dependence on NK cells and suggesting against ADCC as the mechanism of inhibition. In contrast, macrophages were observed to be ingesting opsonized virus particles, implicating ADCP in inhibition of influenza infection (111). Subsequent work demonstrated that neutrophils may be an essential phagocyte that interacts with influenza specific antibody in protection from disease (112).

Another line of evidence showed that depletion of lung phagocytes, i.e., neutrophils and alveolar macrophages, caused uncontrolled growth of influenza in mice, and caused mortality at doses that were sub-lethal to normal mice (112, 113). Passive immunization studies performed in normal mice or neutrophil-depleted mice demonstrated that the effector mechanism responsible for control of influenza infection by phagocytes was likely antibody-dependent, since control of infection was enhanced by passively infused antiserum (112). Consistent with these results, DeLillo and collaborators used humanized Fcγ receptor mice in a passive immunization model to demonstrate that influenza hemagglutinin protein stalk-specific neutralizing human monoclonal antibodies (see Epitope section below) were dependent on interactions with activatory Fcγ receptors for protection from influenza challenge (114). The antiviral mechanisms of this protection were found to likely be dependent on both ADCC (114) and ADCP (115). Passive immunization experiments performed by He and collaborators, and in a second study by DiLillo and collaborators, also demonstrated a requirement for Fc receptor interactions in order to achieve maximal protection with both neutralizing, and non-neutralizing influenza-specific antibodies (74, 116). He and collaborators found that protection was dependent on alveolar macrophages, and that these cells were capable of ADCP of immune complexes formed with influenza virus and non-neutralizing or neutralizing antibodies, suggesting a role for ADCP in the observed protective efficacy of passive immunization.

The contribution of ADCP to protection from influenza infection observed in passive immunization studies supports antibodies capable of ADCP as a desirable outcome of active immunization to protect against influenza infection. A paramount goal for the armamentarium against influenza pandemics is development of a universal vaccine that is efficacious against the high diversity of influenza subtypes and strain variants that result from antigenic drift. Many approaches toward this goal attempt to focus the immune response on highly conserved influenza antigens. Results of testing such candidate vaccine regimens have suggested that ADCP may be an important immune response elicited by antibodies that target conserved influenza epitopes. Using influenza virus like particles (VLPs) as an immunogen to elicit responses to the highly conserved matrix protein 2 (M2), Song and colleagues demonstrated, in mice, that M2 VLP immunes sera induced cross protection across heterologous influenza A viruses including the 2009 H1N1 pandemic virus, as well as the heterosubtypic H3N2 and H5N1 influenza viruses. They also found that the immune sera responsible for this cross-protection required the presence of dendritic cells and macrophages (117). Another group independently identified similar observations and reached similar conclusions. Using a vaccine approach based on the conserved M2e influenza epitope, they showed that passive immunization by M2e-specific antibodies depends on the presence of alveolar macrophages with intact FcγRI and FcγRIII for immune protection (118). The authors of this study point out that the M2 protein is highly expressed on the surface of infected cells, but limited on influenza virions. They therefore hypothesized that the anti-M2e antibodies preferentially target influenza infected cells and eliminate them via ADCC and ADCP prior to virus propagation and release by budding. ADCP of infected cells is an understudied aspect of immune responses to all viral infections, and it should be further investigated in this context. Collectively, these preclinical candidate vaccine studies provide evidence that protection by Fc receptor-dependent processes including ADCP may be broad and stretch across influenza subtypes. However, ADCP activity has also been shown to be a component of the non-cross-reactive immune response induced by the seasonal trivalent influenza vaccine (119), and anti-influenza sera ADCP activity is common in healthy adults (120). It remains unknown how ADCP and other Fc receptor-dependent functions contribute to persistent natural and vaccine-induced responses to seasonal influenza.

While most evidence from studies performed in animal models suggests that phagocytes and ADCP contribute to protective immune responses against influenza virus there may also be aspects of their function that augment influenza infection. For example, inhibition or removal of the ability of phagocytes to produce reactive oxygen species improved the resolution of lung influenza infection in mice (121), and the high affinity allele of FcγRIIa was found to be a risk factor for severe pneumonia during the 2009 A/H1N1 pandemic (32).

### Herpesviruses

ADCP of herpes simplex virus-infected fibroblast by both neutrophils and monocytes has been observed *in vitro* (122), and several studies have implicated ADCP as having a role in immune responses to herpesvirus infections. ADCP was recently identified as a component of the antibody response elicited by vaccination with a cytomegalovirus (CMV) subunit vaccine. Nelson and collaborators investigated the humoral components of reduced risk of CMV acquisition observed in the moderately (~50%) efficacious clinical trial of the CMV glycoprotein gB/MF59 vaccine conducted in postpartum women (123, 124). Consistent with previous observations (125), they found that this vaccine induced only modest neutralizing antibody responses. However, the vaccine-elicited antibodies were demonstrated to bind to the surface of infected cells and mediate robust ADCP of both gB-protein coated beads and fluorescently-labeled whole CMV virions (124). Interestingly, the vaccine elicited high magnitude gB-specific IgG3 responses, which likely contributes to the high levels of ADCP observed as the IgG3 isotype has previously been shown to be superior to IgG1 for virion ADCP in studies performed with HIV-1 (104). The study by Nelson et al. is novel in that it implicates non-neutralizing antibody responses as having an important role in antibody responses against CMV, and therefore may help to open new pathways toward development of highly effective next-generation CMV vaccines.

A genetic link providing evidence of a role ADCP in immune responses to herpesviruses was described for EBV. The low affinity allele of FcγRIIa was found to be enriched in EBV infection and correlated with the expression of the latency protein LMP1, suggesting that this allele may be a risk factor for latent EBV infection (34).

Collectively these studies provide evidence that ADCP contributes to vaccine-elicited and natural immune responses to herpesvirus infection. Due to the prevalence of herpesviruses worldwide, and the need for additional strategies for treatment and prevention, additional research in this area is warranted.

### Other Virus Infections

Although the ADCP has not been comprehensively studied in the context of most virus infections, it may be a common component of antiviral humoral immune responses to diverse types of viruses as supported by the study of respiratory syncytial virus (RSV), ebola virus, human papilloma virus (HPV), foot-and-mouth disease virus (FMDV), severe acute respiratory syndrome coronavirus (SARS-CoV), and West Nile virus (WNV).

For RSV and ebola virus, evidence of ADCP in immune responses is limited to *in vitro* assessment of virus-specific monoclonal antibodies. ADCC and ADCP activity was observed for RSV G protein-specific monoclonal antibodies produced by B cells from healthy (presumably RSV exposed and immune) adults (126). For ebola virus, glycoprotein-specific monoclonal antibodies produced by B cells in response to ebola virus glycoprotein DNA prime and virus-vectored boost vaccination were shown to have ADCP activity (127).

The contribution of ADCP activity to protection from HPV infection was reported for anti-HPV neutralizing monoclonal antibodies. Using passive transfer experiments in mouse models, Wang and collaborators provided evidence that HPV-specific IgG monoclonal antibodies can cross the vaginal epithelial at sites of local disruption, and that this IgG had ADCP activity (128). They propose that ADCP augmented protection against vaginal HPV infection resulting from virus neutralization because protection was less efficacious when passive transfers were performed with F(ab′)_2_ instead of whole IgG, when performed in Fcγ-deficient mice, or with mice depleted of neutrophils and Gr1^+^ macrophages.

FcγRII genotyping of 180 people previously infected with SARS-CoV and 200 region-matched normal donors was used to identify homozygosity for the low affinity allele of FcγRIIa as significantly associated with severe SARS-CoV infection (33). Further evidence supporting a role for ADCP in the immune response that attenuates SARS-CoV infection comes from depletion studies that demonstrated a requirement for infiltrating and tissue resident macrophages, as well as SARS immune sera, for clearance of SARS-CoV from pulmonary cells in a mouse model (129). NK cells were not required, suggesting against ADCC as the immune mechanism involved in the reduction of infection (129). The SARS immune sera use in these experiments had the ability to neutralize SARS-CoV. Thus, this study provides another example of ADCP contributing to the protection mediated by antibodies with the ability to neutralize virus, as described above for HPV and HIV-1. Similar observations have been made for FMDV (129–131). Taken together, these observations suggest the compelling possibility that for many viruses where neutralization has been shown to have the ability prevent or control infection, ADCP or other Fc receptor-dependent effector functions may also contribute and enhance their protective function to an extent that has yet to be fully defined.

For WNV, passive antibody transfer of WNV non-structural protein-1 (NS1)-specific monoclonal antibodies into normal mice and Fcγ receptor knock-out mice demonstrated Fc receptor-dependent protection (132). Protection was maintained in mice lacking only FcγRIII, suggesting that NK cell ADCC was not essential. The authors demonstrated that murine macrophages were able to internalize NS1-expressing target cells in the presence of anti-NS1 antibodies, generating the hypothesis that ADCP contributed to protection against WNV infection in this model system. Interestingly, this study demonstrates a role for ADCP of virus-infected cells in protection, which is understudied when compared to ADCP of whole virus or virus proteins. It is possible that infected-cell and virion ADCP may in some contexts have divergent contributions to antiviral responses as it has been shown that virion ADCP promotes higher replication of WNV and other flaviviruses using *in vitro* cell culture models (133, 134).

## Potential for ADCP to Enhance Viral Infection

ADCP is not always associated with beneficial or protective immune responses. In some cases, ADCP has been demonstrated to promote infection. Termed antibody-dependent enhancement (ADE), this has predominantly been described for viruses from the genus *Flavivirus* including dengue virus, yellow fever virus, Japanese encephalitis virus, and zika virus (135–140). Fc receptor-dependent ADE is a particular concern for infections by dengue virus (141–143). There are four serotypes of dengue virus, and antibodies generated in response to infection with one serotype have variable degrees of cross reactivity to the other three serotypes. Upon re-challenge with the original serotype, antibodies are protective. However, the pre-existing antibody response will augment infection and promote more severe disease upon secondary infection with any of the other serotypes (143). Moreover, ADE resulting from cross reactivity may not be limited to secondary infection with another strain of the same virus; ADE might also occur when the secondary infection is a different but closely related virus. For example, the results of recent studies have suggested that dengue virus-specific antibodies may be capable of enhancing zika virus infection (144), and reciprocally, that zika virus-specific antibodies may be capable of enhancing dengue virus infection (140). These observations have been met with skepticism (145), and additional work will be required to demonstrate that this type of ADE has clinical relevance to dengue and zika incidence and disease. ADE is thought to occur when non-neutralizing antibodies, or suboptimal concentrations of neutralizing antibodies, bind and opsonize virus, and are subsequently internalized by Fc receptor expressing cells via ADCP. Within the cells these immune complexes may perturb normal antiviral immune functions resulting in the virus not being destroyed. Instead, the virus exploits ADCP as a tool to expand access to host cells, resulting in higher infection burden and often stimulating an overt inflammatory response that contributes to disease pathogenesis (143). How do viruses such as dengue subvert the process of ADCP to promote increased infection? One potential mechanism that has been proposed to allow for dengue virus to escape Fc receptor-mediated antiviral signaling is through engaging the inhibitory receptor leukocyte immunoglobulin-like receptor-B1 (LILRB1) (146). Thus, for dengue and other viruses, the outcome of each interaction between antibody-virus immune complexes and phagocytes depends on the balance of activating signals, which are expected to promote ADCP and virus elimination, and inhibitory signals, which have the potential to enhance infection.

In the studies of HIV-1 infection, early papers reported that complement and antibodies at low titers could enhance HIV-1 infection *in vitro* (147–150). Since monocytes and macrophages can be infected with HIV-1, one theory is that antibody-mediated phagocytosis may have caused increased viral infection of these phagocytes. More specifically, enhancement may have been due solely to weakly or non-neutralizing antibodies that remain capable of engaging but not neutralizing HIV-1 virus (151). However, it is notable that no enhancement of infection in the vaccine arm was observed in the VAX003 and VAX004 clinical trials, both of which elicited high levels of non-neutralizing antibodies (152, 153).

As described in a comprehensive review of the topic by Taylor and collaborators (143), there is evidence of ADE for many other viruses including influenza, SARS-CoV, RSV, and ebola virus—for all of which there also exists evidence of ADCP as being part of beneficial antiviral antibody responses. These dichotomous observations emphasize the balancing act faced by the immune system as it attempts to respond to virus infection. Immune responses of sufficient specificity and potency are required to protect against infection and disease, but suboptimal responses can be exploited by pathogens resulting in higher levels of infection and more severe disease. Because of this, ADE remains a concern for the development of vaccines against viruses for which potent neutralizing antibody response cannot be elicited.

## Viral Epitopes Recognized in ADCP

**Figure 3:**
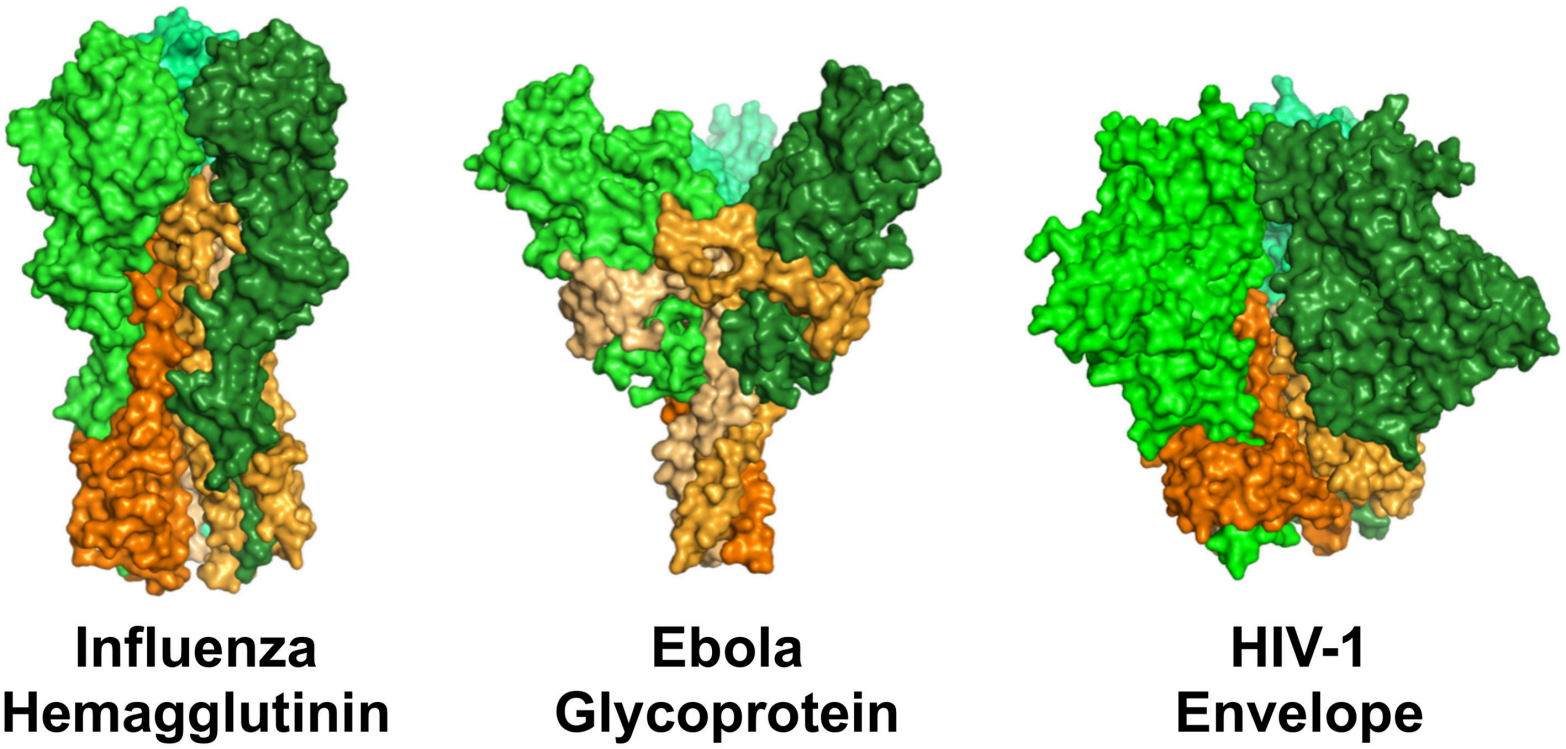
Surface representation of structures of the viral fusion proteins of influenza virus, ebola virus, and HIV-1 that are targets of antiviral antibodies. Perspective is from the side, such that the proteins are standing atop the virus envelope. These envelope proteins share a general structural architecture as trimers of dimeric proteins comprised of a receptor binding domain (head, represented by shades of green) and a viral membrane-bound domain (stem/stalk, represented by shades of orange). Renderings were made in PyMOL using protein data bank ID 3GBN (influenza), 5JQ3 (ebola), and 5FYL (HIV-1).

One of the best examples of how epitope specificity can impact ADCP was described for influenza-specific antibodies. Two distinct epitope regions of the influenza hemagglutinin protein are recognized by influenza-specific antibodies: the immunodominant and antigenically variable head domain; and the more antigenically conserved stalk region (Figure 3). DiLillo and colleagues demonstrated a requirement for antibody–Fcγ receptor interactions for protection by the stalk-specific antibodies, but not for antibodies recognizing the head domain (114). They further demonstrated that the stalk-specific antibodies were capable of activating NK cells for ADCC, and that the protective efficacy of stalk-specific antibodies could be improved with mutations that selectively enhance engagement with FcγRIIa and FcγRIIIa (114). Mullarkey and colleagues followed-up on these insights and identified a similar epitope-dependent dichotomy of anti-hemagglutinin antibody responses in antiviral assays performed with neutrophils as the source of innate effector cells. Using recombinantly-produced chimeric antibodies with mouse Fv and human Fc regions, they demonstrated that stalk-specific, but not head-specific, antibodies induced ADCP, and reactive oxygen production by neutrophils. Similar observations were made for antibodies made both as IgG1, and as IgA, indicating that the epitope dichotomy for interaction with Fc receptors is conserved for Fcγ and Fcα receptors (115). Their use of recombinantly produced antibodies with identical Fc regions confirms that at least for influenza virus, antibody epitope specificity can play an essential role in establishing downstream antiviral effector responses. A subsequent study by DiLillo and collaborators expanded upon their initial observations, and demonstrated that the requirement for Fc receptor involvement for protection *in vivo* was not restricted only to stalk-specific antibodies, but instead was a common feature of antibodies capable or recognizing a breadth of influenza strains. Using mouse models, Fc receptor dependent protection was shown for both broadly neutralizing stalk and head specific antibodies, as well as broadly-binding non-neutralizing head-specific antibodies (116). Thus, there exists a yet to be explained relationship between breadth and Fc receptor-dependence in protective antibody response to influenza. Sera from human donors has the ability to mediate anti-influenza ADCP (119, 120), it will therefore be important for future research to further map the specificities of natural human influenza ADCP antibodies, and to explain the mechanisms underlying the differential functions of the strain-specific head antibodies from multi-strain specific head and stalk antibodies.

A similar epitope dichotomy has been described for ebola virus. The ebola virus envelope glycoprotein is similar to the envelope fusion glycoproteins of influenza and HIV-1 in that they share a common architecture of trimers of dimeric proteins. The dimers are comprised of a distal receptor-binding head region, and a membrane-bound stem/stalk region (Figure 3). The stem/stalk regions are less variable than the head regions, however the head regions are generally regarded as immunodominant. Ebola stalk-specific specific antibodies were demonstrated to be capable of mediating multiple Fc receptor-dependent effector functions including NK cell activation and ADCP by monocytes or neutrophils, while glycan cap-specific mAbs lacked this functionality (154). Thus, it will be interesting to study parallels and differences between broad-binding influenza and ebolavirus stalk-specific ADCP antibodies as the biomolecular interactions underlying the relationships between epitope and function are elucidated.

Diverse HIV-1 epitopes can be targeted for virion phagocytosis, including broadly neutralizing epitopes such as the gp41 MPER (stem region), and gp120 head-region targets such as the CD4 binding site, V2 glycan, and trimer apex. Non-broadly neutralizing epitopes such as the gp41 principal immunodominant domain (stem region), V1/V2 loop and V3 loop (gp120 head region) (104, 155–157) are also targets of ADCP antibodies. The CD4-induced epitopes found on HIV-infected cells are not engaged alone for phagocytosis, but can be engaged synergistically, at least with V2 epitopes, for virus engagement (98). The ability of these epitopes to be engaged for phagocytosis varies by strain, indicating the substantial heterogeneity between viruses. Furthermore, antibodies targeting the same epitope can vary in their phagocytosis potency, emphasizing the fact that the rules of antibody-epitope engagement that allow for ADCP are not completely understood. These may include antibody Fc availability which can be affected by antigen binding valency and angle of approach (158). It is also likely that there are non-neutralizing antigenic targets on the HIV-1 virion surface that are involved in ADCP or other Fc-dependent functions but have not been discovered. Our understanding of the HIV-1 envelope protein (Env) surface is strongly biased toward neutralizing epitopes, since the search for anti-HIV-1 antibodies typically involves the selection and characterization antibodies positive for neutralization. However, these typically make up only a portion of the antibodies that are elicited in response to HIV-1 infection or vaccination, but the non-neutralizing specificities capable of binding and engaging Env are typically not further studied. In addition, monomeric gp140 or gp120 proteins, and even the current generation of stabilized trimeric proteins that are typically used as hooks for antibody selection likely do not fully recapitulate the diversity of natural Env forms on the virion envelope, which may include both functional trimers and non-functional forms of envelope (159). If so, large-scale unbiased antibody screening may identify additional crucial antibody specificities for Fc-mediated effector functions against virions that may be targeted by vaccines or passive immunotherapy. It is important to note that most virion ADCP methods do not differentiate between infectious and non-infectious virion particles–thus, an open question remains as to whether non-neutralizing antibodies that appear to mediate virus ADCP do indeed cause uptake of infectious viruses, or whether they merely engage non-infectious epitopes on defective particles.

Virus-infected cells and virion particles are distinct biological targets of antibody-mediated internalization. The epitope specificities involved for each process may differ, given different epitope exposure on viruses and infected cells. For instance, in the context of HIV-1, the gp41 principal immunodominant domain targeted by antibodies such as 7B2 and F240 is frequently found on virions, but not on infected cells, whereas the converse is true for the conserved region 1 conformational domain targeted by antibodies such as A32 and C11 (104, 160, 161). The majority of studies describing the ADCP response to HIV-1 have been focused on gp120/gp140 proteins or whole virions, thus there is much less known about ADCP of HIV-1 infected cells and the epitopes that may be involved in this process. Additional research in this area is needed for HIV-1 and other viruses.

Comparatively less has been described regarding the fine epitope specificity of ADCP antibodies in the immune responses to other virus infections or vaccination. As a general rule, for ADCP of virions the epitope must be present on the surface of the virions, while for ADCP of infected cells any epitope expressed on the surface of a virus-infected cell may be a potential target. For most viruses limited antibody specificities have been described to mediate ADCP: CMV gB (124) protein, RSV G protein (126), ebola virus glycoproteins (127), HPV L2 protein (128), and WNV NS1 protein (132). In most of these studies, the ADCP activity was identified by testing available antibodies, or antibodies selected for alternative functionality such as neutralization. Therefore, many potential specificities remain unexplored and it is likely that antibodies that target other epitope regions are capable of ADCP activity but have not yet been identified. Overall, there is a dearth of knowledge regarding specific epitopes that can be targeted by ADCP antibodies against most viruses, and more insight into this area may inform new strategies for rational design of vaccines intended to elicit ADCP responses.

## Approaches to Measure Virus ADCP

Virus ADCP is typically measured via a cell-based assay, where the amount of viral target internalized by a phagocyte is quantified. Sample monoclonal or polyclonal antibodies are first pre-mixed with the viral target to form immune complexes. Phagocytes are then introduced for antibody-dependent phagocytosis to occur. Such phagocytes include primary monocytes, macrophages, neutrophils, or corresponding cell lines (104, 162, 163). Phagocytes can be spinoculated (164) with the immune complexes to increase the signal-to-noise ratio, though this is not necessary to achieve detectable signal in the case of antibody-dependent phagocytosis of HIV-1 virions (165). Non-internalized virus particles are then washed off, and the amount of internalized viral target is quantified.

In theory, any method capable of quantifying target particles (e.g., nucleic-acid based RT-PCR, protein-based ELISA, or fluorescence) can be used as the assay readout, as long as internalized virus particles can be separated from extracellular or cell-attached particles. In practice, fluorescence-based approaches are the most popular due to the capability for high-throughput readout via flow cytometry (162). In fluorescence-based approaches, the target particle could be a live fluorescently-labeled virion (104, 120, 166) or a virus-derived protein conjugated to a fluorescent bead (120, 162). Where virions are labeled, this can be done with direct labeling [including viral membrane labeling which would exclude detection of membrane-fused viruses (167)], or internal labeling via co-transfection of genes encoding fluorescent non-structural proteins, resulting in their random incorporation into packaged virus particles (168). Fluorescence labels can also be engineered to report internalization, either by the addition of a non-cell-permeable fluorescence quencher during the wash step (169), or by using a pH-sensitive dye.

It is as yet unclear how results compare between different types of viral targets. Notably, phagocytosis signaling pathways and particle uptake dynamics are impacted by the physical properties of the target, including ligand spacing (170, 171), size (170, 172), shape (173), stiffness (174), and antigen height (175). Since virus particles differ from protein-coated beads in a number of these attributes, it remains unclear how these differences affect Fc-Fc receptor signaling, or how they influence the range of virus epitope-Fab conformations that are capable of mediating phagocytosis. In the case of HIV-1, one group has argued that HIV-1 virus particles cannot undergo ADCP due to insufficient surface ligand density (176). This was based on their observation of undetectable internalization of HIV-1 viruses by HIV-specific antibodies as compared with an anti-phosphatidylserine antibody. However, this result is at odds with our work demonstrating epitope-specific internalization of HIV-1 virus particles using a variety of HIV-specific monoclonal antibodies of different Env specificities (100), as well as polyclonal antibodies from sera and breast milk (165). Since the experimental procedures used by both groups were similar, the reasons for this discrepancy are unclear, and may involve the method of virus preparation.

Moving forward, it will be important to determine how the nature of the immune complex affects phagocytosis. Current assays do not routinely measure the size of the immune complex engulfed, but only the number of intracellular or intravesicular virions, which may be either a single immune complex or an amalgamation of multiple co-phagocytosed viruses. In fact, for many viruses, it is not even clear whether antibody-dependent virion internalization is strictly a process of phagocytosis, or whether other processes including endocytosis and macropinocytosis are involved. It will also be important to determine the signaling outcomes of such phagocytosis in multiple cell types and inflammatory conditions, in order to determine the role of phagocytosis in the recruitment of other effector functions as well as the subsequent development of the local inflammatory response and longer term adaptive immune priming. For highly polymorphic viruses including HIV-1 and influenza, it will also be useful to develop a panel of representative viral strains (fluorescent if necessary) for use as a reproducible measure of the breadth of ADCP activity which can be compared across different settings and clinical trials. Indeed, just as HIV-1 antibody neutralization potency against tier 1 viruses did not necessarily predict neutralization breadth, ADCP breadth may provide surprising clues for the development of vaccines that elicit strong Fc effector function.

In contrast to ADCP of virus particles, methodology for ADCP of virus-infected cells remains underexplored, as most current ADCP assays have been developed to measure internalization of immune complexes formed with virions, or protein-coated beads. Thus, it remains difficult to quantify how antibody epitope specificity and Fc profile contribute toward their potency for ADCP of virus-infected cells. Methodologies to investigate ADCP of virus-infected cells both *in vitro* and *in vivo*, in particular differentiating such events from non-antibody-mediated phagocytosis of virus-infected cells, will also be important to determine whether infected-cell ADCP results in virus inhibition or spread.

In addition to ADCP, trogocytosis is another potential outcome of Fc-dependent interactions between antibodies and virus-infected cells. Two assays developed to measure HIV/SIV-specific ADCC responses have been shown to also detect antibody-mediated interactions between monocytes and virus-infected or protein-coated cells (177, 178). Both assays use fluorescent dyes to label infected or protein-coated target cells. Flow cytometric analysis of cell populations after incubation of these target cells with antiviral antibodies and innate effector cells identified CD14^+^ cells present in the effector cell population as having acquired the fluorescent dye used to label the target cells —indicating transfer of target cell membrane (trogocytosis) and/or ADCP of cells or cell fragments. More recently, an *in vitro* HIV-1 trogocytosis assay has been developed, using flow cytometry to specifically measure the transfer of membrane fragments from a gp120-coated CD4^+^ T cell line to the THP-1 monocytic cell line (179). The relationship between trogocytosis and phagocytosis remains unclear. It may be that these two processes are not mutually exclusive, and that trogocytosis occurs as a result of “aborted phagocytosis” (180). Alternatively, antibody-dependent trogocytosis may represent an alternative mechanism for elimination of target cells (181–183). Presently, the roles of trogocytosis in immune responses to virus infection are not known, and additional research will be needed to determine how this process impacts virus pathogenesis and to further explore potential integrations with ADCP.

## Considerations For Translation of Studies Performed in Animal Models

**Figure 4:**
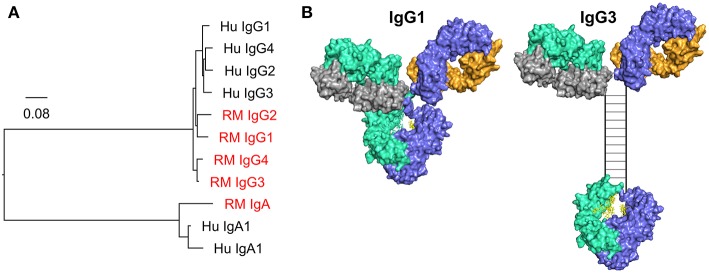
**(A)** Phylogenic tree of human (Hu) and rhesus macaque (RM, red) IgG and IgA C_H_ genes. Scale bar represents the percentage sequence dissimilarity as a measure of phylogenetic distance. **(B)** The human IgG3 hinge region is elongated when compared to IgG1. The hinge region in the hybrid surface/schematic rendering of human IgG3 is drawn to scale, with an estimated hinge length of 80 angstroms, as estimated by Lu and colleagues using small angle x-ray scattering data, and consistent with prior estimates from electron microscopy data (185, 186). Disulfide bonds (*n* = 11) are represented by rungs in the ladder structure (187).

There are significant barriers to the translation of Fc effector functions between animals and humans. The most notable is the difference between the Fc/Fc receptor systems of rhesus, mice, and humans. The differences between the mouse and human Fc/Fc receptor system have been reviewed previously (184). While rhesus and human Fc/Fc receptor systems might be expected to be more similar, there remain substantial differences. Fc differences are apparent when comparing IgA and IgG subclasses between rhesus macaques and humans (Figure 4). Rhesus macaques have only one IgA gene, whereas humans have two IgA genes, IgA1 and IgA2. While both rhesus macaques and humans have four IgG subclasses, rhesus IgGs are not structurally or functionally analogous to human IgG1-4. In fact, rhesus IgG subclasses are genetically more similar to each other than they are to their human homologues (Figure 4A), and the diversity in the germline immunoglobulin genes is higher in rhesus macaques than in humans (188, 189). Structural differences between rhesus and human antibodies are best exemplified by the IgG3 subclass. The human IgG3 C_H_ domain encodes repeats of a hinge region exon, resulting in a hinge that is approximately four times longer (in number of amino acids) than that of human IgG1 (Figure 4B) (190). There is no such exon duplication and hinge elongation in rhesus macaque IgG3 (188). The structure of the hinge dictates the flexibility of the antibody (185), and therefore impacts biological functions and has implications for translation of studies performed in humans and rhesus macaques. For example, vaccine-elicited HIV-specific IgG3 was identified as a correlate of reduced risk of infection in the RV144 clinical trial (87, 89), and *in vitro* studies have demonstrated higher ADCP activity for IgG3 HIV monoclonal antibodies compared to other IgG subclasses (104). Given the differences between human and rhesus IgG3 it is unlikely that preclinical studies performed in the rhesus model would have predicted that vaccine-elicited IgG3 could play a crucial role in reducing the risk of HIV infection as observed in RV144. Caveats such as this must be considered when using animal models.

There are also differences in how human and rhesus macaque antibodies interact with Fc receptors. For instance, rhesus IgG2 and IgG4 retain strong binding to Fc receptors (191, 192), whereas human IgG2 and IgG4 have severely attenuated binding (6). Thus, there is more similarity in Fc receptor interactions and effector functions (191) across rhesus IgG compared to that observed for humans. This suggests that rhesus macaques do not use IgG subclasses to tune antibody responses to the same extent as humans, and highlights the need for caution when comparing subclass profiles in humoral responses across these species. In addition, rhesus macaque Fc receptors are more highly polymorphic than human Fc receptors (189, 192) and the functional implications of many of these polymorphisms have yet to be defined. Importantly, while rhesus and human Fc and Fc receptors can cross-react in binding, antibody binding to FcγRIIa is different between humans and rhesus macaques when comparing antibody Fc mutants, suggesting that antibodies may not behave similarly against human and rhesus macaque FcγRIIa (193), which is commonly engaged for ADCP (162). Despite these differences, our preliminary data indicate that there remains cross-reactivity and some level of functional homology for phagocytosis between humans and rhesus macaques, with similar rank order of phagocytosis activity across species when human IgG and IgA isotypes/subclasses are tested against human or rhesus monocytes and neutrophils (Pollara et al., unpublished observations).

Further studies are required on the interactions between Fc and Fc receptors in different species, their effects on phagocytosis, and the subsequent effects of phagocytosis to determine what findings from preclinical studies can be translated. Passive immunotherapy may be simpler to model since there appears to be functional homology for phagocytosis between human and rhesus systems when human Ig is used. Humanized mice will also be valuable for these types of studies. Specifically, the mouse model developed by Smith and collaborators in the laboratory of Dr. Jeffrey Ravetch is expected to provide the most utility (194). In their model, the mouse Fcγ receptor genes have been deleted and human Fcγ receptor transgenes have been inserted. This results in mice that express functional human Fcγ receptors on the same types of cells and at the same levels as found in humans. The primary limitations of this model are that only human Fcγ receptors are expressed. Thus, Fcα-receptors, FcεRI, Fc receptor neonatal, Type II Fc receptors, and other Fc-binding proteins remain as native mouse forms. Moreover, repeated infusion of human IgG can result the development of anti-human IgG antibodies in these mice (195).

## Conclusions and Future Perspectives

ADCP is an Fc receptor-dependent function of antibodies that is likely common to immune responses elicited by virus infection and in response to vaccination. Importantly, there is substantial evidence that supports ADCP as contributing either to protection from infection, or reduction in disease severity for diverse types of viruses. In most cases, it is likely that ADCP works in tandem with additional Fc-independent and Fc-dependent antiviral activities as part of an effective polyfunctional humoral response. In fact, for many viruses that have been demonstrated to have antibody-dependent correlates of protection it is highly likely that ADCP is involved, but perhaps was not thoroughly explored. Additional investigation into the role of ADCP in protective viral responses, and the specific virus epitopes targeted by ADCP antibodies, may provide insight into strategies for rational vaccine design to elicit these types of antibody responses while avoiding deleterious ADE activity. Additionally, identifying the types of phagocytes and Fc receptors involved in ADCP at sites of virus infection within tissues, throughout the course of infection and virus clearance, remains an understudied aspect of host and virus interactions. Finally, as most ADCP assays used to measure this immune response *in vitro* quantify uptake of virus subunits, virions, and in some cases infected cells, there remains a gap in knowledge regarding the outcome of phagocytosis. Further research into this area could determine if protection by ADCP is dependent on clearance and elimination of virus and virus infected cells, or by potentiating subsequent immune responses via antigen presentation or immune signaling. As our understanding of ADCP grows, it is likely that approaches to successfully leverage this important immune response for improved antiviral immunity will be discovered.

## Author Contributions

MT and JP conceived and wrote the manuscript. KW performed protein modeling for the figures and wrote the manuscript.

### Conflict of Interest Statement

The authors declare that the research was conducted in the absence of any commercial or financial relationships that could be construed as a potential conflict of interest.
